# Association of selected gene polymorphisms with thermotolerance traits in cattle – A review

**DOI:** 10.5713/ab.22.0055

**Published:** 2022-06-24

**Authors:** Dwi Nur Happy Hariyono, Peni Wahyu Prihandini

**Affiliations:** 1 Department of Animal Science, Faculty of Agriculture, Universitas Khairun, Ternate 97719, Indonesia; 2 Beef Cattle Research Station, Indonesian Agency for Agricultural Research and Development, Pasuruan 67184, Indonesia

**Keywords:** Cattle, Genes, Heat Stress, Marker-assisted Selection, Single Nucleotide Polymorphism, Thermotolerance

## Abstract

Thermal stress due to extreme changes in the thermal environment is a critical issue in cattle production. Many previous findings have shown a decrease in feed intake, milk yield, growth rate, and reproductive efficiency of cattle when subjected to thermal stress. Therefore, selecting thermo-tolerant animals is the primary goal of the efficiency of breeding programs to reduce those adverse impacts. The recent advances in molecular genetics have provided significant breeding advantages that allow the identification of molecular markers in both beef and dairy cattle breeding, including marker-assisted selection (MAS) as a tool in selecting superior thermo-tolerant animals. Single-nucleotide polymorphisms (SNPs), which can be detected by DNA sequencing, are desirable DNA markers for MAS due to their abundance in the genome’s coding and non-coding regions. Many SNPs in some genes (e.g., *HSP70*, *HSP90*, *HSF1*, *EIF2AK4*, *HSBP1*, *HSPB8*, *HSPB7*, *MYO1A*, and *ATP1A1*) in various breeds of cattle have been analyzed to play key roles in many cellular activities during thermal stress and protecting cells against stress, making them potential candidate genes for molecular markers of thermotolerance. This review highlights the associations of SNPs within these genes with thermotolerance traits (e.g., blood biochemistry and physiological responses) and suggests their potential use as MAS in thermotolerant cattle breeding.

## INTRODUCTION

Recently, global climate change has had an adverse impact on the adaptability and survivability of farm animals to thermal assault. The elevation of ambient temperature, especially in summer, is a critical issue in the agricultural-related industry. One of the most important effects of thermal stress in the livestock industry is the decrease in cattle productivity and reproductivity. Numerous studies have demonstrated the significant decline in cattle performance when subjected to thermal stress; reduction in feed intake [1,2], milk yield [3,4], growth rate [5,6], and reproductive efficiency [7,8]. Thermal stress, such as heat stress, has also caused economic losses of around $897 million and $369 million annually for dairy and beef cattle industries, respectively [9], pressuring livestock producers to adopt effective preventative measures.

To mitigate the adverse impacts of thermal stress on cattle productivity, some strategies can be implemented, including genetic selection, modification of the environment, and nutritional intervention [10]. Besides, scientific techniques such as cooling systems and sprinkler treatments have been developed to reduce the adverse impact of heat stress on animals [11,12]. It has been reported that within-breed genetic variation exists for thermotolerance, allowing genetic selection for improved thermotolerance and may increasing animal resilience and welfare [13]. Furthermore, animal genetic selection for thermotolerance provides cumulative and permanent solutions at a relatively low cost [14]. To date, genetic selection in beef and dairy cattle is directed to productive traits, such as growth rate, meat production, milk yield, and milk quality. However, a continual selection for productive traits ignoring heat tolerance, results in decreasing heat tolerance [7]. There are considerable evidence that there is a genetic antagonism between productive traits and the specific ability to respond to heat stress [7,14,15]. For instance, selection for milk yield results in a poor response of the animals to heat stress [15]. Therefore, a combined selection for production and thermotolerance under highly modified environments, in both tropical and sub-tropical regions, should be considered. Furthermore, a set of phenotypes, including body temperature (BT) and respiration rate (RR), in combination with measures of indicators of heat tolerance in milk, can be used as an alternative in the genomic selection of heat-tolerant dairy cattle without damaging progress in milk production [14].

In conventional breeding methods, the selection of superior animals with economically important traits related to thermal stress is based on observed phenotypes and information on animal pedigree. However, the advancement of molecular genetics offers several approaches enabling the researchers to improve the accuracy of estimated breeding values and accelerate the achievements by selecting animals based on their genotypes rather than waiting to measure their physical attributes (phenotype) later in life. In molecular breeding methods, marker-assisted selection (MAS) is one of the approaches that allow the identification of specific DNA variations that are associated with particular economic traits like thermotolerance. Due to some advantages, single nucleotide polymorphisms (SNPs) are desirable DNA markers for MAS in livestock breeding. The SNPs are abundant throughout an organism’s genome (both coding and non-coding regions). The SNPs that cause an amino acid substitution in the gene coding regions are called non-synonymous; those that do not are synonymous. In addition, SNPs are more stably inherited than other DNA markers, making them more suited as long-term selection markers [16].

In cattle, several SNPs within some genes (e.g., *HSP70*, *HSP90*, *HSF1*, *EIF2AK4*, *HSBP1*, *HSPB8*, *HSPB7*, *MYO1A*, and *ATP1A1*) have been reported to be associated with thermotolerance traits [17–25]. All the mentioned genes have critical roles in many cellular activities during thermal stress, protecting cells against stress. Furthermore, stress-induced gene expression and activation of heat shock proteins (HSPs) are key indicators of the animal’s response to thermal stress in cellular and molecular activities [26]. Some potential indicators of thermal stress, such as blood biochemistry and physiological responses (BT, RR, and rectal temperature [RT]) were proposed to elucidate the impact of the mentioned genes on thermotolerance in cattle [17,18,27]. BT and RR can be measured using automated phenotyping technologies [28]. Therefore, it is very important to have deep knowledge about the genetic basis of the animal’s response to thermal stress to identify molecular markers for thermotolerance in livestock breeding.

This study discussed some potential candidate genes and their associations with thermotolerance traits in cattle. The genes discussed in this review included *HSP70*, *HSP90*, *HSF1*, *EIF2AK4*, *HSBP1*, *HSPB8*, *HSPB7*, *MYO1A*, and *ATP1A1* (Table 1).

## THERMOTOLERANCE TRAITS IN CATTLE

It is well-known that temperature is one of the significant environmental factors affecting the livestock populations’ health and productivity. Rising global temperatures put heat stress at the center of ever-growing concern regarding livestock populations across tropical and temperate zones [29]. Genetic selection for thermotolerance is likely to be one of the potential strategies to overcome the effect of rising temperatures on cattle productivity. Carabaño et al [30] proposed several measurements as indicators of thermotolerance in animals, including BT, heart rate (HR), RR, and sweating rate (SR). Several biomarkers like blood biochemical parameters have also been proposed as selection criteria to identify thermo-tolerant animals [17]. It has been reported that RT is the best predictor of core BT. The heritability of RT during heat stress is moderate making it possible to use this trait as selection criteria to improve thermotolerance [31]. However, the inheritance of physiological traits, such as RT and HR is still little known, owing the fact that they are extremely labor-intensive to collect, especially in a large-scale selection program. Koltes et al [28] proposed some automated temperature detection devices that can be used to provide real-time temperature data and monitor potential thermal stress in animals. Those devices allow research that can inform producers on how best to manage or select thermo-tolerant animals.

Identification of thermo-tolerant animals is challenging because the responses to heat stress are complex and variable. Besides, there is antagonism between thermotolerance and productive traits, as reported in some dairy cattle populations [7,14,15,32]. For instance, selection for higher milk yield results in a reduced animal’s ability to cope with heat stress [15]. Negative genetic correlations from −0.85 to −0.24 between thermotolerance and productive traits (e.g., milk yield) have been observed in dairy cattle [32]. Therefore, it is important to monitor thermotolerance when selection for productive traits is implemented in production systems influenced by heat stress. Furthermore, there are substantial genetic variations in an individual animal to cope with greater heat loads, with a moderate degree of genotype x environment interaction. This suggests that animals that produce the most in comfort may not be the best animals under heat stress [30]. Consequently, the genetic variation in tolerance to heat stress should be considered in the selection of dairy cattle raised under modified environments in both tropical and sub-tropical regions.

## CANDIDATE GENES FOR THERMOTOLERANCE

### Heat shock protein 70 (*HSP70*)

Heat shock proteins (HSPs) are a group of proteins that protect cells from harmful oxidative stress. The HSPs family consists of HSP110, HSP100, HSP90, HSP70, HSP60, HSP40, HSP10, and small HSP [33]. Among the HSPs, the proteins of molecular mass 70 kDa, termed HSP70, is the most essential molecular chaperone of primary importance to all mammalian cells, which acts by binding to other cellular proteins and facilitating intracellular transport [34]. HSP70 is produced by the *HSP70* gene, characterized by a single exon. The open reading frame of this gene is approximately 1926, and its protein consists of 641 amino acids, of which 92 and 82 are highly basic and acidic amino acids, respectively [35]. Heat shock genes are activated by stressful stimuli, further forming HSPs. The HSP70 expression in heat-stressed dairy animals was found to be the most responsive compared to other HSPs chaperones. Therefore, the *HSP70* expression profile could be beneficial as a biomarker to determine the effect of thermal stress in animals [36].

The *HSP70* gene family in bovine consists of four genes, including *HSP70-1*, *HSP70-2*, *HSP70-3*, and *HSP70-4* [37]. In cattle, both the *HSP70-1* and *HSP70-2* genes are located on chromosome 23 band 22 (the BoLA region). In contrast, the *HSP70-3* gene is located on chromosome 10 band 34, and the *HSP70-4* is localized at chromosome 3 band 13 [38]. Gallagher et al [38] screened bovine genomic DNA for hybridization with the *HSP70* cDNA in humans. They found that bovine *HSP70-1,2* were homologous with human *HSPA1* and *HSPA1L* on chromosome 6p21.3, while bovine *HSP70-3* was homologous with a human *HSP70* gene on chromosome 14q22-q24 and bovine *HSP70-4* was the homolog of the human *HSPA-6,-7* genes on chromosome 1.

**Figure 1:**
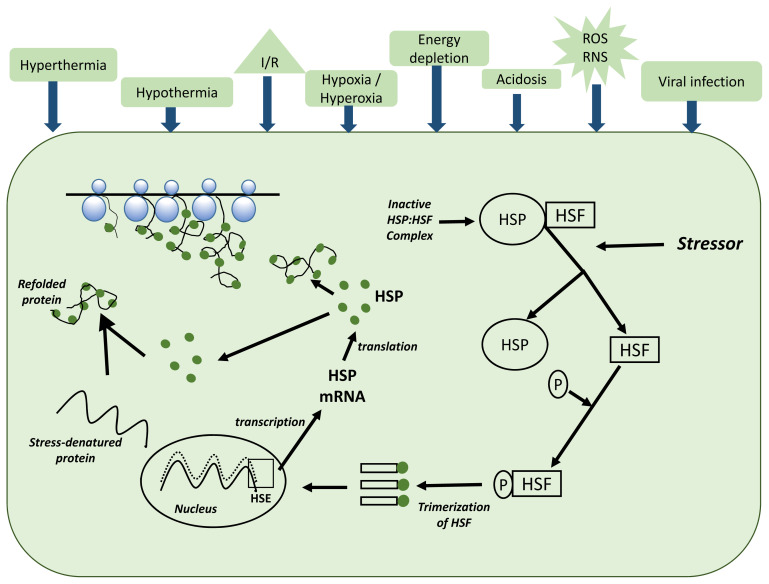
Molecular mechanism for the expression of *HSP70* within a cell, adapted from Kregel [40]. The *HSP70* expression is modulated by some physiological signals like thermal stress, energy depletion, acidosis, and viral infection. These physiological stimuli activate the heat shock factors (HSFs), causing them to seperate from heat shock proteins (HSPs). The HSFs are phosphorylated by protein kinases and form trimers in the cytosol. These HSF trimer complexes enter the nucleus and bind to heat shock elements (HSE) in the promotor region of the *HSP70* gene. HSP70 mRNA is then transcribed and leaves the nucleus for the cytosol, where new HSP70 is synthesized. Proposed mechanisms of cellular protection for HSPs include their functioning as molecular chaperones to assist in the assembly and translocation of newly synthesized proteins within the cell and the repair and refolding of damaged (e.g., stress-denatured) proteins.

Figure 1 shows the molecular mechanism for the expression of *HSP70* within a cell. A cell undergoes molecular changes when exposed to thermal stress. In this regard, most of the HSP family genes, including *HSP70*, have crucial cytoprotective effects and are involved in many regulatory pathways related to cell stress response [33]. Besides the behavioral and physiological responses, thermal stress can induce the *HSP70* expression [39,40]. However, genetic variation among individual animals may cause varying thermal stress coping capacities. Therefore, understanding the physiological systems that are controlled by genes involved in thermoregulation is important to prioritize variants in genomic selection strategies in addition to understanding the modulation of genotype × environment interaction [41].

Xu et al [42] identified 193 differentially expressed genes in peripheral blood samples of Sanhe cattle exposed to severe cold stress (−32°C for 3 h). Out of the 193 candidate genes, the expression of *HSP70* significantly increased after cold exposure (p<0.05). An earlier study conducted by Abbas et al [17] on lactating Holstein cows under heat and cold stress observed a significant association between SNPs in the 5′ flanking region of the *HSP70* gene and blood biochemical parameters. SNPs A-12G and C181T in the *HSP70* gene significantly affected (p<0.05) lactate concentration, while A72G was associated (p<0.05) with lipid peroxide concentration under heat stress conditions, and the same association exists for SNPs A-12G and SNP C131G with dopamine and superoxide dismutase concentrations [17].

Polymorphisms in the *HSP70* gene have also been associated with blood biochemistry in Inner-Mongolia Sanhe cattle, a dual-purpose (milk and meat) cattle breed in the Inner Mongolia Autonomous Region of China [43]. Among 20 SNPs identified in the *HSP70* gene in Sanhe cattle, SNP-42^−^ located in the promoter region and SNPs −105^+^, −181^+^, and −205^+^ localized in the 5′-untranslated region (UTR) have been associated with blood triiodothyronine (T3), while SNP −105^+^ has been associated with blood thyroxine (T4). The SNPs −42^−^ and −205^+^ have been observed to be the causative mutations involved in regulating *HSP70* promoter activity [43]. Amino acid changes from aspartate to tyrosine and from aspartate to histidine due to G>T and G>C substitutions, respectively, at 149th of the amplicon (295 bp) in the *HSP70* gene in Tharparkar cattle have been identified to produce two alleles (alleles A and B with nucleotides T and C, respectively), in which genotype AA was superior in heat tolerance [44]. Also, the identified SNP was novel and has been submitted to National Center for Biotechnology Information (NCBI) with GenBank Accession Number JX966362 for allele A and JX966363 for allele B. Onasanya et al [45] in their earlier study identified twelve SNPs of the *HSP70* gene in four zebu breeds of Nigeria; White Fulani, Sokoto Gudali, Red Bororo, and Ambala. Of the twelve variants, four (C151T, C146T, G90A, and C219A) were unique as they were detected in all the analyzed breeds. The SNP C146T was synonymous, while C151T, G90A, and C219A were non-synonymous mutations, causing changes in the coded proteins from serine to leucine, from alanine to arginine, and from cysteine to threonine, respectively. Regarding the four SNPs, heterozygous animals had lower a heat tolerance coefficient (HTC), suggesting their potential to withstand heat stress than homozygous counterparts [45].

Prihandini et al [46] identified fourteen SNPs in the 5’-UTR of the *HSP70* gene in several cattle breeds in Indonesia (Galekan, Bali, Jabres, Belgian Blue×Peranakan Ongole [PO] cross, Rambon, Madura, and PO). The SNPs included C1036T, G1045A, A1058G, C1069T, G1076A, A1096G, G1117A, A1125C, G1128T, T1134C, G1164T, T1204C, C1255T, and C1262T. Among these SNPs, SNP G1045A, T1134C, and T1204C have been associated with the serum concentration of T3 and IGF-I and body condition [47,48], while the SNP A1125C was associated with calving percentages in crossbred Brahman cows [48]. The SNP G1076A identified by Prihandini et al [46] in Indonesian cattle breeds was also observed in South African Nguni crossbred cattle [49]. Six SNPs in the *HSP70* gene, including G1045A, G1117A, A1125C, G1128T, T1134C, and T1204C, have been identified in some cattle populations from different countries [46,50–52]. Of the six SNPs, SNP G1128T has been associated with cell viability and gene expression of the *HSP70.1* gene in Holstein cattle, in which heterozygous animals showed higher cell viability (p<0.05) and gene expression (p<0.001) compared to homozygous GG [50]. The SNP G1128T was also associated with calving percentages in crossbred Brahman cows [48]. Badri et al [53] found SNP G462T in the coding region of the *HSP70* gene in Chinese Holstein cattle with an amino acid substitution at position 154 from glutamine to histidine, which may influence heat tolerance.

### Heat shock protein 90 (*HSP90*)

HSP90 is a member of the HSPs family, which has an important biological role in protein translocation and regulation of steroid hormone receptors [36]. The HSP90 is a versatile chaperone protein that maintains cellular integrity and homeostasis during heat stress [39]. This protein is considered a highly conserved and essential stress protein expressed in all eukaryotic cells, comprising 1% to 2% of cellular proteins under non-stress conditions [54]. The HSP90 is the regulator for nearly 100 proteins termed client proteins, involved in signal transduction [54]. Among the HSPs, HSP90 and HSP70 are the two important components of the cellular machinery involved in protein homeostasis and participate in nearly every cellular process. In some chaperone activities the two proteins work together in cellular remodeling functions [55] and act as apoptosis modulators, by interfering with the formation of the apoptosome, thereby reducing cell death during heat shock [56].

When the mammalian cells are exposed to external stress, a way to respond to such stress is by releasing some conserved proteins, such as the HSPs family. Among the HSPs, the *HSP90* expression has been associated with the freezing resistance of bull sperm. The higher expression of *HSP90* leads to the higher motility and freezing resistance of sperm after freezing-thawing [57]. *HSP90* expression in bull spermatozoa gradually declined following freezing-thawing process, thereby affecting sperm quality [58]. There are two major cytoplasmic isoforms of HSP90, including the inducible form (HSP90α or HSP90AA1) and the constitutive form (HSP90β or HSP90AB1), which are constituted by the duplication of genes [27,59]. *HSP90AA1* and *HSP90AB1* genes have been mapped to bovine chromosome 21 (with 733 amino acids) and 23 (with 724 amino acids), respectively. The HSP90 isoforms contribute to cellular functions, including signal transduction, protein folding, protein degradation, cell survival, and morphological evolution [60].

Certain polymorphisms in the *HSP90* gene in various breeds of cattle throughout the world have been identified. Onasanya et al [18] identified four SNPs in the exon 3 of the *HSP90* gene in four zebu breeds in Nigeria, i.e., Ambala, Red Bororo, Sokoto Gudali, and White Fulani. The identified SNPs were T116G, G220C, G346A, and G390A. Heterozygous animals showed significantly (p<0.0001) lower BT, RT, RR, and HTC than homozygous animals [18]. The *HSP90AB1* gene polymorphisms have also been detected by Charoensook et al [61] in indigenous Thai breed White Lamphun and Mountainas, as well as a crossbred Holstein Friesian. In their studied populations, a total of nine SNPs were observed, one in the 3’-UTR (g.5435T>C), three in exons 10 (g.4374T>G) and 11 (g. 5007T>C and 5082C>T), and five in introns 8 (g. 4029G>C and 4061G>A), 9 (g.4338T>C), 10 (g.4730A>C), and 11 (g.5248C>T). The SNP g. 5082C>T in exon 11 results in an alanine-to-valine amino acid change. Furthermore, the T allele at SNP T4338C in intron 9 significantly improved heat tolerance (p<0.05) [61]. Interestingly, the SNP g.4338T>C has also been detected in Indian breeds of dairy cattle, Sahiwal and Frieswal, in which TT genotype had significantly (p< 0.01) higher HTC than CT and CC genotypes [62].

Genetic analysis of the *HSP90AA1* gene in Karan Fries (5/8 Holstein Friesian × 3/8 Tharparkar) showed a point mutation, g.1209A>G in exon 3, which produced three genotypes, i.e., AA, AG, and GG. Association analysis for the SNP g.1209A>G showed that homozygous GG had significantly (p<0.01) lower RR, RT, and HTC than homozygous AA and heterozygous AG [27]. A total of five SNPs of the *HSP90AA1* gene were detected in Chinese Holstein cows; one in the promoter (g.−87G>C), three in the coding (g.605A> G, 1662T>G, 2819G>A), and one in 3′-UTR (g.4172A>G) regions [63]. For the g.−87G>C locus, animals having CC genotype had significantly (p<0.01) higher *HSP90AA1* mRNA expression than those having GG genotype in stress conditions [63]. Thus, the SNPs detected in the *HSP90* gene could be potential molecular markers for thermotolerance in cattle breeding.

### Heat shock factor 1 (*HSF1*)

Heat shock factors (HSFs) are the main transcription factors that maintain protein homeostasis (proteostasis) and counteract disturbances by regulating heat shock gene transcription [64]. The HSFs are activated in response to stress factors, such as rising temperature, while inactive in non-stressed cells [65]. The HSFs transiently activate the HSPs transcription by binding to their promoters [64]. The HSPs were first observed in *Drosophila melanogaster* in the early 1960s, as revealed by a “puffing” of genes in the chromosome of recovering cells [66]. The “puffing” has been shown to activate genes encoding the HSPs, which function as molecular chaperones [67]. The HSF family consists of four members in mammals, including HSF1, HSF2, HSF3, and HSF4. Among them, HSF1 is the primary regulator of the heat shock gene transcription in response to stress conditions [68]. The *HSF1* gene was mapped to bovine chromosome 14, and appears to be involved in cardiac health, cell functions, stress responses, and DNA damage repair in mammals [19,69]. Animals with an abundance of the *HSF1* gene expression have a great capacity to cope with heat stress compared to those with lower expression [70]. Consequently, the *HSF1* expression can be used as indicator of thermotolerance in cattle.

Although the importance of the *HSF1* gene as a regulator of the heat shock responses, a few studies have been conducted on identifying the *HSF1* polymorphisms in cattle when compared to the HSPs family [19,71,72]. Sharma et al [73] characterized the 3’-UTR of the *HSF1* gene in Indian cattle breeds, consisting of three SNPs (G1762T, C1811T, and C1983T), and binding sites for several miRNAs. They also noted the abundance expression of most of the miRNAs after heat stress in peripheral blood mononuclear cells of the studied animals, which may affect the function of the *HSF1* gene in response to heat stress. A total of twelve SNPs of the *HSF1* gene were identified in Angus cattle raised in subtropical conditions [72]. Of the twelve, eight were in coding regions with no alteration in the amino acid sequence of the protein, and others were mapped to intron regions with uncharacterized function. Rong et al [19] identified SNP NC_037341.1 g.616087A>G (rs135258919) in the *HSF1* gene in 35 Chinese cattle breeds, which produced three genotypes, AA, AG, and GG. The SNP was associated with annual temperature (T), relative humidity (RH), and temperature-humidity index (THI) (p<0.01). The mutant allele (G) of the SNP was highly detected in animals raised in regions with more prominent heat stress, suggesting that animals carrying allele G (GG and AG genotypes) may be more tolerant to heat stress than those with allele A (AA genotype) [19]. The genetic polymorphisms of the *HSF1* gene were also studied in Chinese Holstein cattle, in which two SNPs (T909C in intron 3 and G4693T in 3′UTR) significantly affected thermotolerance [71]. Based on these SNPs, nine haplotype combinations were constructed. Animals carrying H2H4 haplotype combination (TCTT) showed higher HTC (p<0.05) and lower potassium content in erythrocytes (p<0.01), decreased rate of milk production (p<0.05), and RT (p<0.05) than those carrying H1H3 haplotype combination (TCGG) [71]. Li et al [71] further noted that the 4693-T mutation caused the disruption of microRNA target binding, resulting in the relief of the transcriptional repression that led to the increased *HSF* gene expression [71]. Furthermore, the SNP G4693T was noted to affect the *HSF1* gene expression by influencing the binding of *HSFI* to bta-miR-484 [74]. Li et al [74] further observed another SNP G1451T, in intron 3 of the *HSF1* gene. Still, this SNP was noted to have no effect on thermotolerance.

### Eukaryotic translation initiation factor 2-alpha kinase 4 (*EIF2AK4*)

The alpha subunit of eukaryotic initiation factor -2 (eIF2α) is a molecule involved in regulating protein synthesis initiation in eukaryotes [75]. The eIF2α is phosphorylated by protein kinases, activated in response to various stress conditions; oxidative stress, heme deficiency, osmotic shock, and heat shock [76]. Eukaryotes have four types of eIF2α kinases, including *PKR*, *HRI*, *PERK*, and *EIF2AK4* [20]. Among these, the *EIF2AK4* gene represses the translation of most mRNAs in response to stress-induced signals [77]. The *EIF2AK4* gene is also known as the general control non-derepressible 2 (*GCN2*) gene. This gene is activated in response to viral infection and oxidative stress [78,79]. The activation of the *EIF2AK4* gene also occurs by binding uncharged transfer RNAs (tRNAs) to the HisRS domain of the protein [80].

Heat exposure in mammals can cause oxidative stress and DNA damage resulting in apoptosis [81]. Edea et al [69] detected candidate genes associated with thermo-tolerance and DNA damage repair, including *HSPA4*, *HSF1*, *CMPK1*, and *EIF2AK4*. The *EIF2AK4* gene plays a central role in regulating gene expression in response to the deprivation of amino acids and glucose [82]. Moreover, the *EIF2AK4* gene was noted to activate and mediate cellular response to DNA damaging agents (e.g., UV), viral infections, and nutritional deprivation [83]. A recent study by Wang et al [20] characterized the *EIF2AK4* gene in 35 Chinese cattle breeds and two exotic breeds (Angus [*Bos taurus*] and Burma [*Bos indicus*]). The investigated Chinese cattle breeds were classified into southern cattle, which genetically belonged to indicine cattle, and northern cattle groups, which were genetically classified as taurine cattle. They noted a novel SNP, NC_037337.1 g.35615224 T>G, located in exon 6 of the *EIF2AK4* gene, responsible for a p.Ile205Ser substitution. The frequency of the wild-type allele T (NC_037337.1 g.35615224 T>G) gradually increased from the southern cattle groups to the northern cattle groups, while the mutant allele G showed an opposite pattern. The SNP was also associated with thermotolerance in Chinese cattle, as revealed by the GG genotype, which was generally found in regions with higher AT, RH, and THI [20]. However, the mechanisms by which the *EIF2AK4* gene expression affects cattle thermotolerance must be further investigated. As noted by Jiang and Wek [84], the *EIF2AK4* gene was also associated with resistance to apoptosis due to UV irradiation. Loss of *EIF2AK4* gene significantly increases apoptosis during UV exposure. Furthermore, the *EIF2AK4* gene plays a pivotal role in the integrated stress response (ISR) by regulating sensing starvation [85].

### Heat shock factor binding protein 1 (*HSBP1*)

Heat shock factor binding protein 1 (HSBP1) is a novel and conserved protein containing 76 amino acids and two extended arrays of hydrophobic repeats [86]. The HSBP1 interacts with the oligomerization domain of the HSF1 to suppress HSF1's transcriptional activity in response to heat shock [86, 87]. Besides, the HSBP1 also interacts with HSP70 at a later point in the heat shock response relative to the appearance of HSBP1/HSF1 complexes, inhibiting HSF’s capacity to bind DNA and conversion of the trimer to monomer state [86]. At 1.8 A resolution, the HSBP1 contained crystal structure and amino acid residues 6–53, forming a continuous 11-turn long helix [88]. The *HSBP1* gene acts as a negative regulator of heat shock responses, thereby affecting the survival of animals when subjected to thermal stress [86]. In mice, although the HSBP1 reduction in embryoid bodies (Ebs) leads to increased HSF1 activity, it causes defects in the organization of the germ layers and a reduction in the expression of definitive endodermal markers [87]. In cattle, genetic polymorphisms of the *HSBP1* gene were noted on 930 Chinese Holstein cattle [21]. Consequently, three SNPs (e.g., g.324G> C, g.589C>T, and g.651C>G) formed seven haplotypes and fourteen haplotype combinations. Among the fourteen, the H2H2 haplotype combination had a lower decrease rate of milk yield than the H2H3 haplotype combination (p<0.05). Also, lower potassium content in erythrocytes (PCE) was noted in H2H2 haplotype combination as compared to H2H5 (p<0.05), H4H4 (p<0.05), and H4H5 (p<0.01) haplotype combinations. Moreover, it was noted that the SNP g.651C>G affected thermotolerance in the analyzed cattle; cows carrying GG genotype had lower PCE than CG genotype (p<0.01) [21].

### Heat shock protein family B (small) member 8 (*HSPB8*)

Heat shock protein family B (small) member 8 (HSPB8), also known as HSP22, H11 kinase, or E2IG, is a member of the small HSP family, which is expressed in response to heat shock [89,90]. The HSPB8 is present in the plasma membrane and interacts with the lipid membrane to result in the burial of the tryptophan residues and observable conformational change, thereby interfering with cellular activities such as signal transduction and apoptosis [91]. HSPB8 tends to form small-molecular-mass oligomers and interacts with biological membranes and many different proteins, such as glycolytic enzymes and different protein kinases [92]. Moreover, this protein exists as a monomer *in vitro* and involves in chaperone-like activity as well [89]. The protein also acts as a positive regulator in the PGF2α-induced synthesis of interleukin-6 (IL-6) and vascular endothelial growth factor A (VEGF) in osteoblasts [93]. The *HSPB8* gene has been mapped to bovine chromosome 17 (BTA17q24-25) [22]. In mammals, mutations in the gene encoding HSPB8 can lead to the development of various diseases such as myopathies and neuropathies [90]. The *HSPB8* expression has also been associated with mitochondrial dysfunction that leads to subarachnoid hemorrhage (SAH)-induced early brain injury (EBI) [94]. Verma et al [22] analyzed 108 Sahiwal indigenous cattle in India to reveal SNPs within the *HSPB8* gene and their association with thermotolerance. Two SNPs (e.g., g.507G>A in exon 1 and g.881T>C in intron 1) were noted, but only SNP g.507G>A significantly affected heat tolerance in the studied cattle. For g.507G>A locus, animals with GA genotype had significantly lower (p<0.01) RR, RT, and HTC than those with GG genotype. Therefore, the GA genotype of SNP g.507G>A of the *HSPB8* gene may be advantageous in selecting heat-tolerant animals [22].

### Heat shock protein family B (small) member 7 (*HSPB7*)

The HSPB family is one of the most diverse families within the group of HSP families [95]. The HSPB7 is a member of the HSPB, which is preferentially expressed in cardiovascular, skeletal muscle, and adipose tissue [96]. In obese rats, the *HSPB7* mRNA expression increases in skeletal muscle and brown and white adipose tissues [97]. The *HSPB7* gene appears in oligomeric and dimeric forms (approximately 17 kDa and 40 kDa, respectively) composed of 18.6-kDa monomers [98]. Unlike HSPB1 and HSPB5, that chaperoned heat unfolded substrates and kept them folding competent, HSPB7 did not support refolding [95]. In cattle, the overexpression of *HSPB7* improved the H_2_O_2_-induced oxidative stress in adipocytes via increasing the abundance of NFE2 like bZIP transciption factor 2 (NFE212) and its downstream target genes heme oxygenase-1 (HMOX1) and NADH quinone oxidoreductase 1 (NQO1). Knockdown of HSPB7 markedly inhibited the expression of NFE2L2, HMOX1, and NQO1 and further exacerbated H_2_O_2_-induced oxidative stress [99]. In humans, HSPB7 knockdown promoted osteogenic differentiation of human adipose-derived stem cells (hASCs) via activation of the ERK1/2 signaling pathway [100]. Zeng et al [23] explored 774 individuals representing 32 Chinese indigenous cattle breeds to detect the polymorphism in the *HSPB7* gene. Consequently, one SNP NC_037329.1: g.136054902 C>G was found, of which the allele C was dominant in northern cattle groups. In contrast, allele G was dominant in southern indicine cattle groups. The SNP was associated with AT, RH, and THI (p<0.01). Animals carrying the allele G had higher T, RH, and THI. Thus, SNP NC_037329.1: g.136054902 C>G might benefit heat-tolerant cattle breeding [23].

### Myosin-1a (*MYO1A*)

Myosin-1a (*MYO1A*) gene is a candidate gene associated with skin pigmentation in cattle [69]. As reported previously, skin pigmentation is highly related to the BT regulation [101,102]. In the enterocyte microvillus, the MYO1A was considered the most abundant actin-based motor protein interacting with the apical membrane via a highly basic C-terminal tail domain [103]. The MYO1A belongs to one of eight monomeric, membrane-binding, and actin-based motor protein class I myosins, which are highly expressed in vertebrates [104]. It is also a mechanoenzyme previously thought to be located exclusively in the intestinal epithelium [105]. Furthermore, MYO1A plays an important role in the apical membrane movement and structural stability [106] by powering the sliding of the apical membrane along with microvillar actin bundles [107] and regulating membrane-cytoskeleton adhesion, which enables the apical membrane to resist deformation [108].

In cattle, an association of the *MYO1A* gene polymorphisms with thermotolerance was first described by Jia et al [24] on 1,072 animals from 34 Chinese indigenous cattle breeds, and Angus and Indian zebu. Their study found four SNPs within the *MYO1A* gene, including rs208210464, rs110123931, rs209999142, and rs135771836. A missense mutation of rs209999142 resulted in an amino acid change from phenylalanine to serine. All the identified SNPs were significantly associated with environmental parameters, including T, RH, THI, and SR. Besides, Hap 1/1 constructed based on the four SNPs was advantageous in selecting heat-tolerant animals [24]. Cao et al [109] reported a novel SNP (rs209559414 or NC_037332.1 g.56390345 A>G) within the *MYO1A* gene in Chinese indigenous cattle. This SNP was capable of resulting in an amino acid substitution from isoleucine into valine. The frequency of wild allele A decreased gradually from northern cattle (a temperate monsoon climate) to southern cattle (a subtropical monsoon climate), while that of mutant type allele G showed the opposite pattern. A significant association between genotypes for the SNP rs209559414 and climatic conditions (AT, RH, THI, and average annual sunshine hours [100-cloudiness]) was noted [109].

### ATPase Na+/K+ transporting subunit alpha 1 (*ATP1A1*)

*ATP1A1* gene is a well-known gene involved in regulating BT during heat stress in cattle [25,110–112]. The *ATP1A1* gene has been mapped to bovine chromosome 3, consisting of 22 introns and 23 exons [113]. This gene encoded the alpha-1 chain of Na^+^/K^+^-ATPase, an integral membrane protein of a heterodimeric enzyme, which mediates cutaneous vasodilation during heat stress by interacting with nitric oxide synthase (NOS) [114,115]. This protein also has an important role in membrane permeability by coupling the transport of three Na^+^ ions outward and two K^+^ ions inward [116]. The Na^+^/K^+^-ATPase consists of four isoforms of an α subunit (α1–α4), three isoforms of a β subunit (β1–β3), and FXYD proteins [117]. The four α ATPases include ATP1A1, ATP1A2, ATP1A3, and ATP1A4, encoding for α1, α2, α3, and α4 protein subunits, respectively [118]. Among the α ATPases, the ATP1A1 was highly expressed in erythrocytes and peripheral nerves [115]. It has been reported that the *ATP1A1* mRNA expression of dairy cows under heat stress was higher than those under optimal temperature [110].

Numerous research has been undertaken on various breeds of cattle to determine the association between *ATP1A1* gene polymorphisms and thermotolerance. Liu et al [25] observed two SNPs, −14103G>A in exon 14 and −14242C>T in intron 14 of the *ATP1A1* gene in Holstein cows, which were significantly associated with HTC and RR (p<0.01). Animals with AC genotype were the most tolerant coping with heat stress. A year later, Liu et al [110] discovered a novel synonymous mutation (C2789A) in exon 17 of the *ATP1A1* mRNA in Holstein dairy cows, significantly associated with heat resistance. Due to this SNP, cows carrying the CC genotype showed significantly higher heat tolerance than those carrying the CA genotype (p<0.05) [110]. This SNP was also identified in Vrindavani and Tharparkar cattle of India using polymerase chain reaction single-strand conformation polymorphism and DNA sequencing and associated with heat resistance. Association analysis for this SNP showed that animals with genotype CC showed higher HTC and lower RT than CA and AA genotypes in both breeds [112]. Furthermore, the TT genotype at T27008243C locus in Sahiwal and the AA genotype at 27008223 locus in Karan Fries cows were the most favorable genotypes for heat tolerance [111]. In Cholistani cattle, genetic variant (BB) of the *ATP1A1* gene was found to have significant (p<0.05) effect on vaginal temperature [119]. The polymorphism of the *ATP1A1* gene has also been associated with productive traits like feed intake in European beef cattle breeds [120].

## CONCLUSION AND FUTURE PERSPECTIVES

There is a significant association of SNPs in *HSP70*, *HSP90*, *HSF1*, *EIF2AK4*, *HSBP1*, *HSPB8*, *HSPB7*, *MYO1A*, and *ATP1A1* genes with thermotolerance in various breeds of cattle (both dairy and beef cattle), implying their potential uses as molecular markers in breeding schemes. These molecular markers may benefit both management and breeding decisions to select thermal-tolerant cattle. Since thermal stress is a critical issue in cattle production, mainly due to its deleterious impact on feed intake, milk yield, growth rate, and reproductive efficiency, knowledge of these candidate genes regulating thermal stress can be valuable in providing MAS in cattle breeding. Identification of SNPs in stress-related genes has been proved to generate beneficial molecular data in farm animals. It has been proposed that genomic selection is a promising approach to accelerate genetic gain for thermotolerance because young bulls and heifers can be selected based on their genomic estimated breeding value (GEBV) [32,121]. The higher the young bulls genotyped, the smaller the marginal cost of the additional GEBV is. However, the main challenge in developing a GEBV is the size of the reference population [122]. The accuracy of genomic prediction increases in line with the increased size of the reference population. In dairy cattle, genomic predictions for heat thermotolerance have been conducted using reference populations from Holstein and Jersey cattle genotyped for 632,003 SNPs with an accuracy of 0.39 to 0.57 in Holsteins, and 0.44 to 0.61 in Jerseys [32]. This suggests that genomic selection is an excellent alternative to improve thermotolerance in cattle. Furthermore, emerging technologies in molecular genetic techniques and genome editing, such as clustered regularly interspaced short palindromic repeat (CRISPR)/CRISPR-associated endonuclease Cas9 (Cas9), may pave the way for novel approaches, allowing introduction of site-specific gene modifications. Finally, incorporating molecular information into breeding programs will benefit industry, scientists, and breeders to develop thermo-tolerant cattle and improve the accuracy of traditional selection methods.

## Figures and Tables

**Table 1:** Effects of SNPs in some genes on thermotolerance in cattle

Gene	SNP	GenkBank accession number	Location	Amino acid substitution	Breeds analyzed	Associated trait	Reference
*HSP70*	A-12G	AY149618.1	5′ Flanking Region	None	Holstein cattle	LA, DA,and SOD	[17]
	C181T	AY149618.1	5′ Flanking Region	None	Holstein cattle	LA	[17]
	A72G	AY149618.1	5′ Flanking Region	None	Holstein cattle	LPO	[17]
	C131G	AY149618.1	5′ Flanking Region	None	Holstein cattle	DA and SOD	[17]
	−42^−^ (C/T)	AY149618.1	Promoter	None	Inner-Mongolia Sanhe cattle	Blood triiodothyronine (T3)	[43]
	−105^+^(G/T)	AY149618.1	5′-UTR	None	Inner-Mongolia Sanhe cattle	Blood triiodothyronine (T3) and thyroxine (T4)	[43]
	−181^+^(C/T)	AY149618.1	5′-UTR	None	Inner-Mongolia Sanhe cattle	Blood triiodothyronine (T3)	[43]
	−205^+^(C/T)	AY149618.1	5′-UTR	None	Inner-Mongolia Sanhe cattle	Blood triiodothyronine (T3)	[43]
	G149T	NM_174550.1	Coding region	Aspartate to Tyrosine	Tharparkar cattle	RT, ART, HTC, and RR	[44]
	G149C	NM_174550.1	Coding region	Aspartate to Histidine	Tharparkar cattle	RT, ART, HTC, and RR	[44]
	C146T	NM_174550.1	Exon 1	None	Nigerian zebu: White Fulani, Sokoto Gudali, Red Bororo, and Ambala	HTC	[45]
	C151T	NM_174550.1	Exon 1	Serine to Leucine	Nigerian zebu: White Fulani, Sokoto Gudali, Red Bororo, and Ambala	HTC	[45]
	G90A	NM_174550.1	Exon 1	Alanine to Arginine	Nigerian zebu: White Fulani, Sokoto Gudali, Red Bororo, and Ambala	HTC	[45]
	C219A	NM_174550.1	Exon 1	Cysteine to Threonine	Nigerian zebu: White Fulani, Sokoto Gudali, Red Bororo, and Ambala	HTC	[45]
	G1128T	U09861	Promoter region	None	Holstein cattle	Cell viability, *HSP70* expression	[50]
*HSP90*	T116G	AC_000178.1	Exon 3	Threonine to Histidine	Red Bororo cattle	BT, RT, RR, and HTC	[18]
	G220C	AC_000178.1	Exon 3	Arginine to Serine	Sokoto Gudali	BT, RT, RR, and HTC	[18]
	G346A	AC_000178.1	Exon 3	Serine to Leucine	Ambala	BT, RT, RR, and HTC	[18]
	G390A	AC_000178.1	Exon 3	Aspartate to Tyrosine	White Fulani	BT, RT, RR, and HTC	[18]
*HSP90AB1*	T4338C	NW001494158	Intron 9	None	White Lamphun and Mountainas	HTC	[61]
	T4338C	NW001494158	Intron 9	None	Sahiwal and Frieswal	HTC	[62]
	C5248T	NW001494158	Intron 11	None	White Lamphun and Mountainas	HTC	[61]
	C1787061T	Not mentioned	Intron 10	None	Sahiwal	RT	[123]
*HSP90AA1*	g.1209A>G	AC_000178.1	Exon 3	None	Karan Fries	RR, RT, and HTC	[27]
	g.−87G>C	AC_000178.1	Promoter region	None	Chinese Holstein	*HSP90AA1* mRNA expression	[63]
*HSF1*	g.616087A>G	NC_037341.1	Coding region	Valine into Alanine	Chinese cattle	T, RH, THI	[19]
	T909C	NC_*007312.2*	Intron 3	None	Chinese Holstein	PCE	[71]
	G4693T	NC_*007312.2*	3’-UTR	None	Chinese Holstein	HTC, *HSF1* expression	[71]
	G1451T	NC_007311.3	Intron 3	None	Chinese Holstein	None	[74]
*EIF2AK4*	g.35615224 T>G,	NC_037337.1	Exon 6	Isoleucine to Serine	Chinese cattle, Burma, Angus	T, RH, and THI	[20]
*HSBP1*	g.324G>C	NC_007316.3	Intron 1	None	Chinese Holstein	None	[21]
	g.589C>T	NC_007316.3	Intron 1	None	Chinese Holstein	None	[21]
	g.651C>G	NC_007316.3	Exon 2	None	Chinese Holstein	PCE	[21]
*HSPB8*	g.507G>A	AC_000174.1	Exon 1	None	Sahiwal	RR, RT, and HTC	[22]
	g.881T>C	AC_000174.1	Intron 1	None	Sahiwal	None	[22]
*HSPB7*	g.136054902 C>G	NC_037329.1	Coding region	Alanine to Glycine	Chinese cattle, Angus, Indian zebu	T, RH, and THI	[23]
*MYO1A*	rs208210464	NC_037332.1	Exon region	None	Chinese cattle, Angus, Indian zebu	T, RH, THI, and SR	[24]
	rs110123931	NC_037332.1	Exon region	None	Chinese cattle, Angus, Indian zebu	T, RH, THI, and SR	[24]
	rs209999142	NC_037332.1	Exon region	Phenylalanine to Serine	Chinese cattle, Angus, Indian zebu	T, RH, THI, and SR	[24]
	rs135771836	NC_037332.1	Intron region	None	Chinese cattle, Angus, Indian zebu	T, RH, THI, and SR	[24]
	g.56390345 A>G	NC_037332.1	Coding region	Isoleucine into Valine	Chinese cattle, Angus, Burma	T, RH, THI, and SR	[109]
*ATP1A1*	−14103G>A	NC_007301.3	Exon 14	None	Holstein cattle	HTC and RR	[25]
	−14242C>T	NC_007301.3	Intron 14	None	Holstein cattle	HTC and RR	[25]
	C2789A	NC_007301.3	Exon 17	None	Holstein cattle	Heat resistance	[110]
	C2789A	NC_007301.3	Exon 17	None	Vrindavani and Tharparkar	HTC and RT	[112]
	T27008243C	Not mentioned	Not mentioned	Not mentioned	Sahiwal	RR	[111]
	A27008223G	Not mentioned	Not mentioned	Not mentioned	Karan Fries	RR	[111]

SNP, single-nucleotide polymorphism; *HSP70*, heat shock proteins 70; LA, lactate concentration in blood; SOD, superoxide dismutase concentration in blood; LPO, lipid perioxide concentration in blood; DA, dopamine concentration in blood; RT, rectal temperature; ART, average rectal temperature; HTC, heat tolerance coefficient; RR, respiration rate; ARR, average respiration rate; BT, body temperature; T, annual temperature; RH, relative humidity; THI, temperature humidity index; SR, average annual sunshine hours (100-cloudiness); PCE, potassium content in erythrocytes; *HSF1*, heat shock factors 1; *EIF2AK4*, eukaryotic translation initiation factor 2-alpha kinase 4; *HSBP1*, heat shock factor binding protein 1; *MYO1A*, myosin-1a; *ATP1A1*, ATPase Na+/K+ transporting subunit alpha 1.
